# Prone Position in Mechanically Ventilated COVID-19 Patients: A Multicenter Study

**DOI:** 10.3390/jcm10051046

**Published:** 2021-03-03

**Authors:** Richard Vollenberg, Philipp Matern, Tobias Nowacki, Valentin Fuhrmann, Jan-Sören Padberg, Kevin Ochs, Katharina Schütte-Nütgen, Markus Strauß, Hartmut Schmidt, Phil-Robin Tepasse

**Affiliations:** 1 Department of Medicine B, Gastroenterology, Hepatology, Endocrinology, Clinical Infectiology, University Hospital Muenster, 48149 Muenster, Germany; p.matern@outlook.de (P.M.); Tobias.Nowacki@ukmuenster.de (T.N.); Valentin.Fuhrmann@ukmuenster.de (V.F.); Kevin.Ochs@ukmuenster.de (K.O.); Hartmut.Schmidt@ukmuenster.de (H.S.); 2 Department of Medicine, Gastroenterology, Marienhospital Steinfurt, 48565 Steinfurt, Germany; 3 Department of Cardiology I-Coronary and Peripheral Vascular Disease, Heart Failure, University Hospital Muenster, 48149 Muenster, Germany; jan-soeren.padberg@ukmuenster.de (J.-S.P.); Markus.Strauss@ukmuenster.de (M.S.); 4 Department of Medicine D, Nephrology, University Hospital Muenster, 48149 Muenster, Germany; Katharina.Schuette-Nuetgen@ukmuenster.de; 5 Department of Cardiology, Sector Preventive Medicine, Health Promotion, Faculty of Health, School of Medicine, University Witten/Herdecke, 58095 Hagen, Germany

**Keywords:** prone position, COVID-19 ARDS, lung compliance

## Abstract

Background: The prone position (PP) is increasingly used in mechanically ventilated coronavirus disease 2019 (COVID-19) acute respiratory distress syndrome (ARDS) patients. However, studies investigating the influence of the PP are currently lacking in these patients. This is the first study to investigate the influence of the PP on the oxygenation and decarboxylation in COVID-19 patients. Methods: A prospective bicentric study design was used, and in mechanically ventilated COVID-19 patients, PP was indicated from a partial pressure of oxygen in arterial blood (P_aO2_)/fraction of inspired oxygen (F_IO2_) ratio of <200. Patients were left prone for 16 h each. Pressure levels, F_IO2_, were adjusted to ensure a P_aO2_ greater than 60 mmHg. Blood gas analyses were performed before (baseline 0.5 h), during (1/2/5.5/9.5/13 h), and after being in the PP (1 h), the circulatory/ventilation parameters were continuously monitored, and lung compliance (LC) was roughly calculated. Responders were defined compared to the baseline value (P_aO2_/F_IO2_ ratio increase of ≥15%; partial pressure of carbon dioxide (P_aCO2_) decrease of ≥2%). Results: 13 patients were included and 36 PP sessions were conducted. Overall, P_aO2_/F_IO2_ increased significantly in the PP (*p* < 0.001). Most P_aO2_/F_IO2_ responders (29/36 PP sessions, 77%) were identified 9.5 h after turning prone (14% slow responders), while most P_aCO2_ responders (15/36 PP sessions, 42%) were identified 13 h after turning prone. A subgroup of patients (interval intubation to PP ≥3 days) showed less P_aO2_/F_IO2_ responders (16% vs. 77%). An increase in P_aCO2_ and minute ventilation in the PP showed a significant negative correlation (*p* < 0.001). LC (median before the PP = 38 mL/cm H_2_O; two patients with LC >80 mL/cm H_2_O) showed a significant positive correlation with the 28 day survival of patients (*p* = 0.01). Conclusion: The PP significantly improves oxygenation in COVID-19 ARDS patients. The data suggest that they also benefit most from an early PP. A decrease in minute ventilation may result in fewer P_aCO2_ responders. LC may be a predictive outcome parameter in COVID-19 patients. Trial registration: Retrospectively registered.

## 1. Background

Severe acute respiratory syndrome coronavirus 2 (SARS-CoV-2) is a new virus first identified in December 2019 in Wuhan, China. It can lead to severe pneumonia and respiratory failure (i.e., coronavirus disease 2019 (COVID-19)). Since its identification, the virus has been spreading rapidly across the world. In March 2020, the WHO declared COVID-19 as a pandemic. In February 2021, over 110 million confirmed cases and over 2.4 million confirmed deaths across 215 countries were counted. Mild courses of the disease were observed in 81% of patients, while severe disease courses were found in 14%, and 5% of patients became critically ill and were admitted to an intensive care unit (ICU) [1]. The reasons for admission to an ICU are usually dyspnoea, tachypnea, and hypoxemia, with radiological evidence of pulmonary infiltrates [2,3]. SARS-CoV-2 patients can develop acute respiratory distress syndrome (ARDS). ARDS is a type of respiratory failure characterized by poor oxygenation and pulmonary infiltrates [4]. Currently there is no evidence proven in disease-specific medical treatment available. Current intensive care treatment for COVID-19 ARDS is mostly in line with ARDS recommendations. In patients with early disease, therapy with remdesivir can be evaluated [5,6]. Corticosteroids in critically ill patients can reduce mortality [7]. In patients with severe ARDS and no response to conservative ARDS therapy, VV-ECMO therapy can be evaluated (e.g., P_aO2_/FIO2 < 70 mmHg). For this purpose, ECMO therapy should be evaluated early in the course of disease in patients with severe respiratory failure [8]. Since the 1970s, the prone position (PP) has been used in ARDS patients to treat severe hypoxemia [9,10]. PP means repositioning the patient 180° from the supine position. The PP changes the respiratory mechanics with a reduction in the pleural pressure gradient, a reduction in tidal hyperinflation, ventilation-associated lung damage, and mobilization of secretions [11]. The PP is increasingly used in COVID-19 patients with mild-to-severe ARDS. However, studies on the influence of the PP in SARS-CoV-2 patients are currently lacking. This study investigated the influence of the PP on oxygenation, decarboxylation, and the further course of the disease in COVID-19 ARDS patients.

## 2. Methods

After approval by the institutional human ethics committee (2020-245-f-S; 04/2020), adult patients with a positive polymerase chain reaction (PCR) SARS-CoV-2 nasopharyngeal swab were identified, as previously described [9]. Most patients had a viral load quantification (Ct value at diagnosis, <30), and some patients had only a positive PCR result. In this bicentric, retrospective study, COVID-19 patients admitted to Münster University Hospital (*n* = 38) and to Hospital Steinfurt (*n* = 34) were screened from February to May 2020. ARDS was defined according to the WHO/Berlin definition [12]. Included patients showed bilateral SARS-CoV-2 typical infiltrates on chest X-ray or computed tomography, patients with respiratory failure due to heart failure or volume overload were excluded, and moderate-to-severe oxygenation impairment had to be present. ARDS severity was defined by the ratio of arterial oxygen tension to fraction of inspired oxygen (P_aO2_/F_IO2_). Mild ARDS was defined as 200 mmHg < P_aO2_/F_IO2_ ≤ 300 mmHg (positive end-expiratory pressure (PEEP) or continuous positive airway pressure (CPAP) ≥ 5 cmH_2_O), moderate ARDS as 100 mmHg < P_aO2_/F_IO2_ ≤ 200 mmHg (PEEP ≥ 5 cmH_2_O), and severe ARDS as P_aO2_/F_IO2_ ≤ 100 mmHg (PEEP ≥ 5 cmH_2_O) [11]. All included patients met the WHO severe acute respiratory infection (SARI) definition (respiratory infection with a history of fever, cough, onset within the past 10 days, and required hospitalization) [13]. In 13 patients with moderate or severe ARDS, the PP was performed due to severe hypoxemia (Figure S1). In the following, PP sessions were evaluated independently of the patients (*n* = 36 PP sessions). The PP was performed in patients with a partial pressure of oxygen in arterial blood (P_aO2_)/fraction of inspired oxygen (F_IO2_) ratio of ≤200 in both hospitals. The abdominal positions were performed in both study centers with respect to the same protocol.

Patients were sedated and mechanically ventilated under pressure control (respirators: Evita 4, Dräger, Lübeck, Germany; C3, Hamilton Medical, Bonaduz, Switzerland). No PP was performed in ARDS patients with a contraindication (abdomen apertum, spinal instability, increased intra-abdominal pressure, threatening cardiac arrhythmias, or overt shock). Patients with veno–arterial/veno–veno extracorporeal membrane oxygenation (VA-/VV-ECMO) therapy were excluded from the study for the duration of the ECMO therapy (*n* = 3 patients, 6 PP sessions). Pressure levels and positive end-expiratory pressure (PEEP) were adjusted to individual pressure volume relationships and the fraction of inspired oxygen (F_IO2_) to ensure a P_aO2_ greater than 60 mmHg. PEEP was set according to the ARDS network F_IO2_/PEEP table (higher PEEP/lower F_IO2_) [9]. A target tidal volume of 6 mL/kg ideal body weight was used in both study centers. The patients were left in the PP for 16 h each. The decision to repeat the PP was made individually for each patient depending on the response to the therapy and the overall clinical condition. Patients received continuous sedation and analgetic therapy. The ventilator settings were not changed 30 min before the PP. Arterial blood gas analyses (ABL800 FLEX, Radiometer, Krefeld, Germany) were performed before (baseline, 0.5 h), during (1, 2, 5.5, 9.5, and 13 h), and after (1 h) the PP. The circulatory and ventilator settings were continuously monitored (PEEP, peak pressure (P_Max_), tidal volume, and lung compliance). The ventilation parameters were left unchanged in the first hour of the PP, except for F_IO2_, PEEP, and P_Max_. The P_aO2_/F_IO2_ ratio (mmHg) during the PP sessions (1, 2, 5.5, 9.5, and 13 h) were used to assess oxygenation for the study period.

Patients treated with the PP were classified as either P_aO2_ or partial pressure of carbon dioxide (P_aCO2_) responders. The P_aO2_/F_IO2_ responders were defined as having a ≥15% increase in the P_aO2_/F_IO2_ ratio (mmHg) [14], while the P_aCO2_ responders were defined as having a decrease of ≥2% P_aCO2_ compared to the baseline value (0.5 h before the PP). P_aO2_/F_IO2_ and P_aCO2_ responders were determined at 1, 2, 5.5, 9.5, and 13 h in the PP. The identified P_aO2_/F_IO2_ responders were divided into fast responders (P_aO2_/F_IO2_ >15% in the first 2 h during the PP) and slow responders (P_aO2_/F_IO2_ >15% after 5.5/9.5 h in the PP) [15,16,17].

The lung compliance (LC) was calculated using the following formula: LC = tidal volume (V_T_)/pressure difference (∆ p) = V_T_/(P_Max_ − PEEP) [18]. A normal LC is approximately 200 mL/cm H_2_O.

In this study, data are given as the median and the range. To compare changes in the supine/prone position, a non-parametric one-way ANOVA for repeated measures (Kruskal–Wallis test) was used. Dunn’s multiple comparison post hoc test was used to compare pairs of time points. Correlations of at least ordinally scaled parameters were examined using Spearman’s rank correlation, while correlations of nominally/metrically scaled parameters were performed using the eta correlation coefficient. Calculations were performed using statistical software (SPSS, IBM SPSS Statistics, New York, USA). Differences with a *p*-level less than 0.05 were considered statistically significant.

## 3. Results

The study included 13 COVID-19 patients with ARDS from Münster University Hospital and Marienhospital Steinfurt. Characterization of the patient population is shown in Table 1. All patients had suspected or confirmed bacterial superinfection, and all patients received antibiotic therapy. The median time period from intubation to PP was 0 days, interquartile range (IQR) 0; 2 (Table 2). The number of PP sessions per patient ranged from 1 to 6, and 7 of the 13 patients died within 28 days after the last PP (28 day mortality, equal ICU mortality and equal hospital mortality). The last nasopharyngeal swab before the first pp is shown (Table 2). There was a significant correlation between viral load in deep nasopharyngeal swabs and 28 day mortality (eta coefficient, *r* = 0.950; *p* = 0.01; *n* = 7).

### 3.1. Prone Position Responder: Oxygenation and Decarboxylation

**Figure 1:**
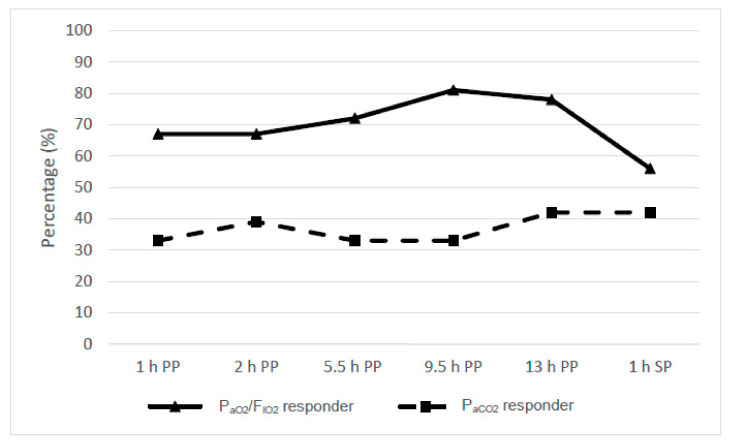
Partial pressure of oxygen in arterial blood/fractional inspired oxygen concentration (P_aO2_/F_IO2_) responder (≥15% improvement in P_aO2_/F_IO2_) and P_aCO2_ responder (≥2% improvement in P_aCO2_) during the prone (PP) and supine (SP) positions. h, hour.

**Figure 2:**
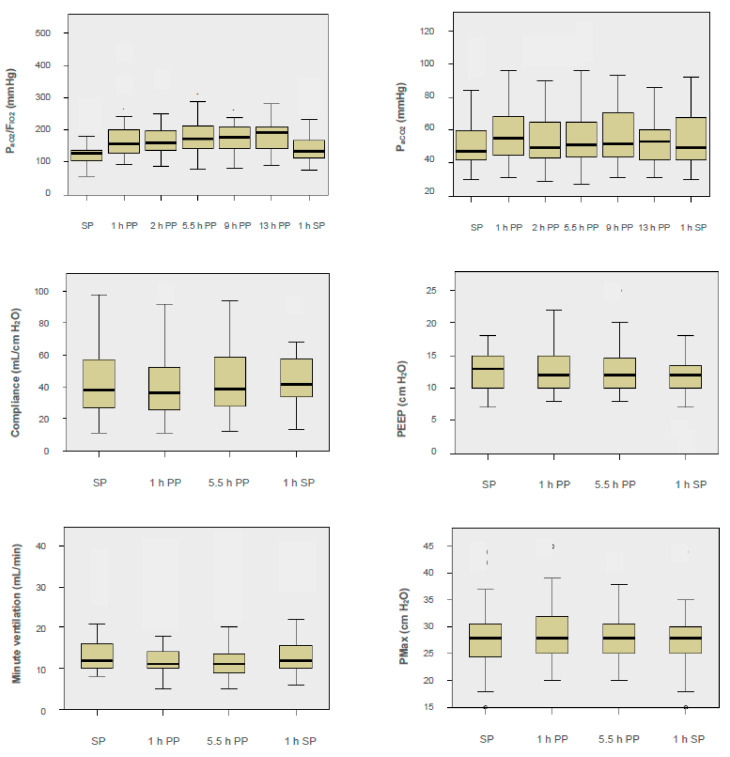
P_aO2_/F_IO2_, P_aCO2_, lung compliance, minute ventilation, peak inspiratory pressure (P_Max_), and positive end-expiratory pressure (PEEP) before and during the prone and supine positions.

Considering the P_aO2_/F_IO2_ ratio after 1 h in the PP, responders were identified in 24/36 (67%) PP sessions (cutoff value: >15% increase in P_aO2_/F_IO2_) (Table 2 and Table 3 and Figure 1). The median improvement of oxygenation (P_aO2_/F_IO2_) was 38.4% [26%; 95%]. The number of PP sessions that had an improvement in oxygenation (P_aO2_/F_IO2_ >15%) continued to increase up until 9.5 h in the PP. Considering the entire period in the PP, 29/36 (81%) responders were identified: 24/36 fast responders, 5/36 slow responders, and 7/36 nonresponders were identified. After turning prone, a significant median improvement (responders and nonresponders) in P_aO2_/F_IO2_ was achieved at the 1, 2, 5.5, 9.5, and 13 h time points (*p* < 0.001; Figure 2). The maximum increase in the P_aO2_/F_IO2_ ratio of the responders was observed after 5.5 h in the PP (median 58.3% [31%; 95%]). In 3/13 patients, the interval from intubation to the first PP was three days or more (3, 9, and 16 days). Considering this subgroup, oxygenation improved significantly (cutoff: ≥15%) only in 1/6 PP sessions (16% vs. 77%).

Subjects treated with PP sessions were classified as either P_aO2_ or P_aCO2_ responders (Table 2 and Table 3A and Figure 1 and Figure 2). A significant decrease in P_aCO2_ was observed in 12/36 (33%) PP sessions (1 h). The median decarboxylation of the responders improved by 4.5% [−27%; −3%]. Most P_aCO2_ responders were identified after 13 h in the PP (42%). This corresponds to three PP sessions with a slow improvement in decarboxylation. The maximum improvement in decarboxylation was seen in the PP after 5.5 h (median −4.5% [−26%; −9%]) (Figure 1). One hour after turning in the supine position, a persistent increase in P_aCO2_ was observed in 15/36 (42%) sessions. At this time point, the responders showed a median decrease of 11% [−25%; −9%]. Overall, the P_aCO2_ in patients showed no significant change (*p* = 0.809; Figure 2).

### 3.2. Invasive Ventilation of COVID-19 Patients in the Prone Position

In 8/36 (22%) PP sessions, a PEEP reduction was possible (1 and 5.5 h in the PP) (Table 3 and Figure 2). After 1 h in the PP, the median PEEP reduction was 14.2% [−22%; −8%]; after 5.5 h, the median reduction was 13.8% [−21%; −8%]. A reduction in the peak pressure (P_max_) was possible after 1 h in the PP in 15/36 cases (42%; median −3.9% [−14%; −3%]) and after 5.5 h in 14/36 cases (39%; median −7.4% [−12%; −6%]). After supine rotation, a persistent reduction in the peak pressure was observed in 12/36 cases (33%; median −22.6% [−27%; −10%]). The maximum of the median PEEP and peak reduction could be reached 1 h after turning to the supine position. Moreover, 25/36 (69%) after 1 h versus 27/36 (75%) after 5.5 h showed a reduction in minute ventilation (median −15.86 [−23%; −6%] versus median −19 [−32%; −8%]). In the supine position (after 1 h), there was a persistent reduction in minute ventilation in 21/36 cases (58%). The maximum reduction of minute ventilation was observed at 5.5 h in the PP.

This study also showed a significant negative correlation between the percentage increase in P_aCO2_ and the percentage change in minute ventilation in the PP (Spearman’s rank correlation: *r* = −0.616, *p* ≤0.001, *n* = 36). There was no significant correlation between the minute ventilation and the PEEP (Spearman’s rank correlation: *r* = −0.09, *p* = 0.601, *n* = 36) or the P_Max_ (Spearman’s rank correlation: *r* = −0.188, *p* = 0.271, *n* = 36) in the PP sessions. 

### 3.3. Lung Compliance in COVID-19 Patients Correlates with 28 Day Survival

The LC (∆ p = V_T_/(P_Max_ − PEEP)) was calculated 0.5 h before the PP, 1 and 5.5 h during the PP, and 1 h after rotation in the supine position again (Figure 2). In terms of median, an LC of 38 mL/cm H_2_O [26 mL/cm H_2_O; 58 mL/cm H_2_O] was found before the patient was turned prone (Figure 2). One hour after rotation, the median LC was 36 mL/cm H_2_O [25 mL/cm H_2_O; 53 mL/cm H_2_O], which was 39 mL/cm H_2_O [27 mL/cm H_2_O; 59 mL/cm H_2_O] after 5.5 h. After a further turn to the supine position (1 h), the median LC of 42 mL/cm H_2_O [33 mL/cm H_2_O; 58 mL/cm H_2_O] was unchanged. Therefore, it can be concluded that in terms of the median, there was a reduced LC in the COVID-19 patients. LC did not change significantly in patients during proning (*p* > 0.05). Two patients had an LC of >80 mL/cm H_2_O (84 mL/cm H_2_O, 98 mL/cm H_2_O). The LC (1 h before the PP) showed a significant correlation with patient survival (28 day mortality: Eta coefficient, *r* = 0.418; *p* = 0.011).

## 4. Discussion

Since 1974, the PP has been the subject of numerous studies in ARDS patients. Most patients placed in the PP exhibit mild-to-dramatic improvements in oxygenation, regardless of lung injury categorization. There are multiple factors that may influence oxygenation in the PP by reducing ventilation and perfusion mismatches, the intrapulmonary shunt, and ventilator-induced lung injury (VILI) [14]. These changes are due to a reduction in dependent atelectasis and pleural pressure gradient [19], to alveolar recruitment [20], to homogeneous ventilation [21], and to a reduction in lung compression by the heart and abdomen [22,23,24].

The PP in the treatment of therapy-resistant oxygenation disorders in ARDS patients may be most effective when initiated early during the exudative phase. At this point, congestive and compressive atelectasis develop, whereas in the intermediate phase of ARDS (more than one week), fibrosis and type II cell hyperplasia are more prevalent [25,26,27]. In this study, the interval from intubation to the first PP was three days or more (3, 9, and 16 days) in 3/13 patients. Considering this subgroup, oxygenation improved (cutoff: ≥15%) only in 1/6 (16%, 1 h PP) PP sessions versus 23/30 (77%) when the interval from intubation to PP was less than three days. These results suggest that ARDS patients with COVID-19 also benefit most from an early PP (less than three days from intubation to prone ventilation). The 28 day mortality after the last PP session was 54% (7/13 patients died). In studies since 2010, the overall rates of mortality in ARDS patients in an ICU were shown to be 38% and the 28 day mortality to be 30% [28]. In a subgroup of ARDS with H1N1 viral infection, a mortality of 36–38% has been reported [29]. In comparison to H1N1, as well as a meta-analysis of ARDS patients, there was a significantly increased mortality rate in our SARS-CoV-2 patient cohort.

### 4.1. Prevalence of Positive Oxygenation Response in COVID-19 Patients

In this bicentric, retrospective observational study, 29/36 PP sessions (81%) showed significant improvement in the P_aO2_/F_IO2_ ratio (≥15%, 9.5 h PP). The proportion of responders to prone positioning reported in different studies varies depending on the selected cutoff values for oxygenation improvement, the selected time points (comparison supine position with PP), and the number of subjects sampled. In most studies, cutoff values for an increase in P_aO2_ or P_aO2_/F_IO2_ of 10–20 mmHg or an increase of 10–20% have been used to define responders to prone positioning [14]. In approximately 20% of studies, an improvement in oxygenation was found in less than 70% of patients. In the majority of previous studies (approximately 50%), an improvement of 70–85% was achieved in patients. Moreover, 30% of the studies found a significant improvement in oxygenation in ≥90% of ARDS patients in the PP [14]. The time required to improve oxygenation in the PP differs in ARDS patients. In spontaneously breathing patients with COVID-19 with severe hypoxemic respiratory failure, the PP when awake is associated with improved oxygenation [30,31,32,33,34]. In this study, compared to the baseline, positive responders were identified after 1 h in 24/36 (67%) PP sessions. This is a low rate of responders compared to previous ARDS studies (80% of the studies had >70% responders). When considering all control points, most responders were identified 9.5 h after turning prone (29/36; 78%). This is the average number of responders compared to other studies. In 5/36 PP sessions, there was a slow increase in oxygenation (17% slow responders). The majority of patients experienced a rapid improvement in oxygenation in 1 h. These observations are consistent with previous ARDS data [35,36]. On average, P_aO2_ increases of 23–78 mmHg and improvements in oxygenation of 34–62% are seen in responders to the PP [14]. In this study, P_aO2_/F_IO2_ responders showed a maximum increase 5.5 h after rotation to the PP. The median oxygenation improved by 58.3% [31%; 95%]. In comparison to previous studies, there was a good-to-above average improvement in oxygenation in P_aO2_/F_IO2_ responders.

### 4.2. Alveolar Ventilation in COVID-19 Patients: Prevalence of Positive Decarboxylation Response

The effect of the PP on P_aCO2_ in ARDS patients has been described inconsistently in clinical studies. Various factors have been identified to have an influence on the elimination of CO_2_ in the PP. Patients in the PP can experience both increased and reduced elimination of CO_2_. Most observational studies have shown no improvement in P_aCO2_ clearance in patients in the PP. These results are surprising, because in the PP, an improvement in the ventilation/perfusion ratio of the lungs (V_A_/Q) and a reduced intrapulmonary shunt from recruitment leads to improved physiological dead-space fraction [37]. An increase in P_aCO2_ in the PP may depend on a decreased alveolar ventilation and a relative change in lung perfusion [15]. A reduced elasticity of the thorax in the PP with a consecutively reduced breathing volume and minute ventilation can lead to an increase in P_aCO2_ in pressure control ventilation [4]. An increase in CO_2_ elimination appears to be associated with an increase in the net recruitment of lung sections [38]. An increased dead-space fraction is a feature of the early phase of ARDS. It has been shown that an increased dead-space fraction is associated with an increased risk of death [39]. A decrease in P_aCO2_ in the PP is predictive of improved outcome in ARDS [15,40].

Several investigators have classified patients treated with the PP in P_aCO2_ responders and nonresponders. Cutoff values for a P_aCO2_ decrease differ from 1 to 2 mmHg [15,16,17]. One hour after turning in the PP, in 12/36 (33%) PP sessions, a significant P_aCO2_ response (P_aCO2_ decrease of ≥2%) was detected. Most of the values above the cutoff were found in the PP after 13 h (15/36, 42%). Compared to previous ARDS studies (53–54% responders), fewer responders were identified in SARS-CoV-2 patients [12,13]. After 1 h in the PP, responders had a median P_aCO2_ reduction of 4.5% [−27%; −3%]. The maximum P_aCO2_ reduction in P_aCO2_ responders was observed after 5.5 h in the PP (median −12% [−26%; −9%]). These findings are in agreement with previous ARDS data [15].

Regarding PEEP, P_Max,_ and minute ventilation, no significant differences could be found in the PP. There was a significant negative correlation between the percentage increase in P_aCO2_ and the percentage change in minute ventilation in the PP (*p* < 0.001). A reduction in minute ventilation can result in an increase in P_aCO2_ in the PP in ARDS patients [4]. There was no significant correlation between minute ventilation and PEEP (*p* = 0.601) or P_Max_ (*p* = 0.271) in the PP. The results of this study suggest that a decrease in minute ventilation in ARDS patients with COVID-19 leads to fewer P_aCO2_ responders.

### 4.3. Significant Correlation between Lung Compliance and Survival in COVID-19 Patients

LC is the change in lung volume for a given pressure. Before turning to the PP, the patients showed a significantly reduced LC (median 38 mL/cm H_2_O [26 mL/cm H_2_O; 58 mL/cm H_2_O]). During the PP, there was no significant change in LC (*p* = 0.804). The compliance of the respiratory system was similar to other cohorts of COVID-19 patients and to ARDS not related to COVID-19 [18,41,42]. LC was >80 mL/cm H_2_O in two patients, confirming the thesis that there are some COVID-19 patients with severe ARDS and relatively high LC [43]. In our study, there was a significant positive correlation between LC and 28 day survival. To the best of our knowledge, this is the first study to show a significant correlation between LC and survival in ventilated COVID-19 patients. These results should be further examined and confirmed in subsequent, larger studies.

## 5. Conclusions

COVID-19 patients showed significant improvement in the P_aO2_/F_IO2_ ratio in PP sessions (17% slow responders). Our data suggest that they benefit most from an early PP. Compared to previous ARDS studies, fewer P_aCO2_ responders (42%) were identified in SARS-CoV-2 patients. P_aCO2_ increase and minute ventilation in the PP showed a significant negative correlation (*p* < 0.001). The results suggest that a decrease in minute ventilation in COVID-19 patients may result in fewer P_aCO2_ responders. The viral load and the 28 day mortality correlated significantly (*p* = 0.01). Patients showed a reduction in the median LC, and interestingly, there were some patients with relatively high LC, confirming that there are some COVID-19 patients with severe ARDS and relatively high LC. LC and 28 day survival showed a significant positive correlation (*p* = 0.01), indicating that LC may be a predictive parameter for disease course and outcome in COVID-19 patients. These results should be further examined and confirmed in subsequent, larger studies.

## Figures and Tables

**Table 1:** Characterization of the severe acute respiratory syndrome coronavirus 2 (SARS-CoV-2) acute respiratory distress syndrome (ARDS) patient population, including cardiovascular risk factors and other pre-existing conditions, pre-medication, and co-infections.

*n* = 13	Characterization of the Patient Population			
	**Min.**	**Max.**	Median (Q1; Q3)	Avg. (± SD)
Age (years)	45	78	63 (51; 69)	61 (± 10.2)
Body weight (kg)	72	140	80 (76; 98)	88 (± 19.8)
BMI (kg/m^2^)	22	36	25 (25; 30)	27 (± 4.4)
				
Cardiovascular risk factors		Pre-medication		
	*n* (%)			*n* (%)
Arterial hypertension	6 (46%)	Immunosuppressive therapy		0 (0)
Diabetes mellitus	0 (0%)	Antihypertensive therapy		6 (46%)
Nicotine abuse	4 (30.6%)	ACE-inhibitor		1 (7.7%)
Male gender	12 (92.3%)	AT1R-antagonists		3 (23.1%)
		Insulin therapy		0 (0%)
Other pre-existing conditions		Oral diabetes therapy		0 (0%)
	*n* (%)	Systemic steroids therapy		0 (0)
Cardiovascular diseases	5 (38.5%)	Local steroids therapy		0 (0)
Cerebrovascular diseases	1 (7.7%)	Antiviral medication		2 (15.4%)
COPD	0 (0%)			
Bronchial asthma	0 (0%)	Co-infections		
Other pulmonary diseases	0 (0%)			*n* (%)
Immunosuppressive disease	2 (15.4%)	Bacterial superinfection		13 (100%)
Comorbidity	12 (92.3%)	Mycotic superinfection		3 (23.1%)
Medication before illness	12 (92.3%)	Antibiotic therapy (ICU)		13 (100%)

Q1, quartile 1; Q3, quartile 3; Avg., average; SD, standard deviation; BMI, body mass index, COPD, chronic obstructive pulmonary disease, ACE-inhibitor, angiotensin-converting enzyme inhibitor; AT1R-antagonists, angiotensin II receptor type 1 antagonists; ICU, intensive care unit.

**Table 2:** Demographic and clinical characteristics of the patients.

Patient Number	Age	Gender	Charlson Comorbidity Index (CCI)	Severity of ARDS: P_aO2_/F_IO2_ Ratio on The Day of Prone	Intubation to Prone Ventilation in Days	Number of Prone Ventilation Sessions	P_aO2_/F_IO2_ Responder (1 h PP)	P_aCO2_ Responder (1 h PP)	Viral Load: Ct Value (RT-PCR)	PEEP Reduction (1 h PP)	P_Max_ Reduction (1 h PP)	Minute Ventilation Reduction (1 h PP)	28 Day Mortality after Last PP
1	65	Male	4	102	0	4	4/4	0/4	27.4	1/4	2/4	3/4	Died
2	68	Male	3	128	9	3	1/3	1/3	35.6	1/3	0/3	2/3	Survived
3	50	Male	1	145	0	2	2/2	0/2	22.7	0/2	1/2	1/2	Died
4	60	Male	3	97	0	2	2/2	0/2	0	0/2	1/2	2/2	Survived
5	64	Male	3	92	0	3	3/3	2/3	36.8	2/3	3/3	1/3	Died
6	63	Male	3	61	0	1	1/1	1/1	-	1/1	0/1	1/1	Died
7	63	Male	3	99	0	2	2/2	1/2	-	2/2	0/2	2/2	Survived
8	70	Male	4	200	3	2	0/2	1/2	35.1	0/2	2/2	1/2	Survived
9	45	Male	0	88	0	3	0/3	1/3	0	1/3	1/3	2/3	Survived
10	77	Male	4	124	1	5	4/5	3/5	-	1/5	3/5	4/5	Died
11	58	Male	2	127	16	1	0/1	0/1	-	0/1	1/1	0/1	Survived
12	78	Male	4	115	1	2	1/2	0/2	-	0/2	0/2	2/2	Died
13	48	Female	1	58	1	6	4/6	1/6	-	0/6	1/6	4/6	Died

ARDS, acute respiratory distress syndrome; P_aO2_/F_IO2_, partial pressure of oxygen in arterial blood/fractional inspired oxygen concentration; PP, prone position; P_aCO2_, partial pressure of carbon dioxide; PEEP, positive end-expiratory pressure; P_max_, peak inspiratory pressure.

**Table 3:** Changes in the P_aO2_/F_IO2_ ratio and P_aCO2_ in response to prone ventilation sessions, changes in the PEEP, P_Max_, and minute ventilation in response to prone ventilation sessions.

P_aO2_/F_IO2_						
	1 h PP	2 h PP	5.5 h PP	9.5 h PP	13 h PP	1 h SP
Responder (≥15%)	24/36 (67%)	24/36 (67%)	26/36 (72%)	29/36 (81%)	28/36 (78%)	20/36 (56%)
M [Q1; Q3] increase (responder)	38.4% [26%; 95%]	42.6% [23%; 84%]	58.3% [31%; 95%]	40.9% [28%; 67%]	48% [36%; 81%]	46.6% [30%; 62%]
Avg. (± SD) increase (responder)	69.7% (± 67%)	62.8% (± 58%)	68.7% (± 52%)	55.8% (± 49%)	65.7% (± 50%)	52% (± 32%)
PP1	9/13 (69%)	12/13 (92%)	11/13 (85%)	11/13 (85%)	12/13 (92%)	11/13 (85%)
PP2	8/11 (73%)	6/11 (55%)	8/11 (73%)	9/11 (82%)	8/11 (73%)	4/11 (36%)
PP3	4/6 (67%)	3/6 (50%)	3/6 (50%)	5/6 (83%)	5/6 (83%)	5/6 (83%)
PP4	3/3 (100%)	1/3 (33%)	3/3 (100%)	3/3 (100%)	2/3 (66%)	0/3 (0%)
PP5	0/2 (0%)	2/2 (100%)	1/2 (50%)	1/2 (50%)	1/2 (50%)	0/2 (0%)
PP6	0/1 (0%)	0/1 (0%)	0/1 (0%)	0/1 (0%)	0/1 (0%)	0/1 (0%)
**P_aCO2_**						
	**1 h PP**	**2 h PP**	**5.5 h PP**	**9.5 h PP**	**13 h PP**	**1 h SP**
Responder (≥2%)	12/36 (33%)	14/36 (39%)	12/36 (33%)	12/36 (33%)	15/36 (42%)	15/36 (42%)
M [Q1; Q3] increase (responder)	−4.5% [−27%; −3%]	−9% [−21%; −5%]	−12% [−26%; −9%]	−9% [−21%; −4%]	−11% [−21%; −7%]	−11% [−25%; −9%]
Avg. (± SD) increase (responder)	−11.8% (± 13.1%)	−13.3% (± 11.8%)	−16.5% (± 12.6%)	−15.5% (± 17%)	−16.3% (± 14.6%)	−18.9% (± 16%)
PP1	6/13 (46%)	7/13 (54%)	7/13 (54%)	7/13 (54%)	6/13 (46%)	7/13 (54%)
PP2	1/11 (9%)	2/11 (18%)	1/11 (9%)	2/11 (18%)	3/11 (27%)	4/11 (36%)
PP3	3/6 (50%)	3/6 (50%)	2/6 (33%)	1/6 (17%)	2/6 (33%)	3/6 (50%)
PP4	1/3 (33%)	1/3 (33%)	0/3 (0%)	0/3 (0%)	1/3 (33%)	0/3 (0%)
PP5	1/2 (50%)	1/2 (50%)	2/2 (100%)	2/2 (100%)	2/2 (100%)	1/2 (50%)
PP6	0/1 (0%)	0/1 (0%)	0/1 (0%)	0/1 (0%)	1/0 (100%)	0/1 (0%)
**PEEP**						
	**1 h PP**		**5.5 h PP**			**1 h SP**
Reduction	8/36 (22%)		8/36 (22%)			15/36 (42%)
M [Q1; Q2] decrease	−14.2% [−22%; −8%]		−13.8% [−21%; −8%]			−19.9% [−30%; −12%]
Avg. (± SD)	−16.2% (± 9%)		−15.1% (± 7.8%)			−21.4% (± 12.2%)
PP1	2/13 (15%)		2/13 (15%)			4/13 (31%)
PP2	1/11 (9%)		3/11 (27%)			4/11 (36%)
PP3	3/6 (50%)		1/6 (17%)			3/6 (50%)
PP4	2/3 (67%)		2/3 (67%)			2/3 (67%)
PP5	0/2 (0%)		0/2 (0%)			2/2 (100%)
PP6	0/0 (0%)		0/2 (0%)			0/1 (0%)
**P_Max_**						
	**1 h PP**		**5.5 h PP**			**1 h SP**
Reduction	15/36 (42%)		14/36 (39%)			12/36 (33%)
M [Q1; Q2] decrease	−3.9% [−14%; −3%]		−7.4% [−12%; −6%]			−22.6% [−27%; −10%]
Avg. (± SD)	−7.7% (± 6.6%)		−9.3 (± 5.9%)			−18.8% (± 9.5%)
PP1	4/13 (31%)		5/13 (38%)			4/13 (31%)
PP2	6/11 (55%)		4/11 (36%)			2/11 (18%)
PP3	2/6 (33%)		3/6 (50%)			4/6 (67%)
PP4	2/3 (67%)		1/3 (33%)			1/3 (33%)
PP5	1/2 (50%)		1/1 (50%)			1/2 (50%)
PP6	0/1 (0%)		0/1 (0%)			0/1 (0%)
**Minute ventilation**						
	**1 h PP**		**5.5 h PP**			**1 h SP**
Reduction	25/36 (69%)		27/36 (75%)			21/36 (58%)
M [Q1; Q2] decrease	−15.8% [−23%; −6%]		−19% [−32%; −8%]			−18.5% [−35%; −6%]
Avg. (± SD)	−18.7% (± 17.3%)		−22.4% (± 17.5%)			−21.5% (± 10.6%)
PP1	7/13 (54%)		8/13 (62%)			9/13 (69%)
PP2	10/11 (91%)		9/11 (82%)			5/11 (45%)
PP3	4/6 (67%)		6/6 (100%)			2/6 (33%)
PP4	2/3 (67%)		3/3 (100%)			3/3 (100%)
PP5	1/2 (50%)		0/2 (0%)			1/2 (50%)
PP6	1/1 (100%)		1/1 (100%)			1/1 (100%)

P_aO2_/F_IO2_, partial pressure of oxygen in arterial blood/fractional inspired oxygen concentration; h, hour; PP, prone position; SP, supine position; M, Median; Q1, quartile 1; Q3, quartile 3; Avg., average; SD, standard deviation; P_aCO2_, partial pressure of carbon dioxide. PEEP, positive end-expiratory pressure; P_max_, peak inspiratory pressure; h, hour; PP, prone position; SP, supine position; M, Median; Q1, quartile 1; Q3, quartile 3; Avg., average; SD, standard deviation.

## Data Availability

The datasets used and/or analyzed during the current study are available from the corresponding author on reasonable request.

## Supplementary Materials

The following are available online at https://www.mdpi.com/2077-0383/10/5/1046/s1, Figure S1: Study flowchart prone position (PP) in coronavirus disease 2019 (COVID-19) patients. ICU, intensive care unit; ARDS, acute respiratory distress syndrome.

## Abbreviations

SARS-CoV-2	severe acute respiratory syndrome coronavirus 2
ICU	intensive care unit
ARDS	acute respiratory distress syndrome
PP	prone position
PP sessions	prone position sessions
VA-/VV-ECMO	veno–arterial/veno–veno extracorporeal membrane oxygenation
PEEP	positive end-expiratory pressure
LC	lung compliance
V_T_	tidal volume
∆p	pressure difference
VILI	ventilator-induced lung injury
