# Supplementary material for: Prone Position in Mechanically Ventilated COVID-19 Patients: A Multicenter Study
Source: J Clin Med. 2021 Mar 3;10(5):1046. doi: 10.3390/jcm10051046 (PMC7959453; doi:10.3390/jcm10051046)

**Supplementary Figure S1.** Study flowchart prone position (PP) in coronavirus disease 2019 (COVID-19) patients. ICU, intensive care unit; ARDS, acute respiratory distress syndrome.

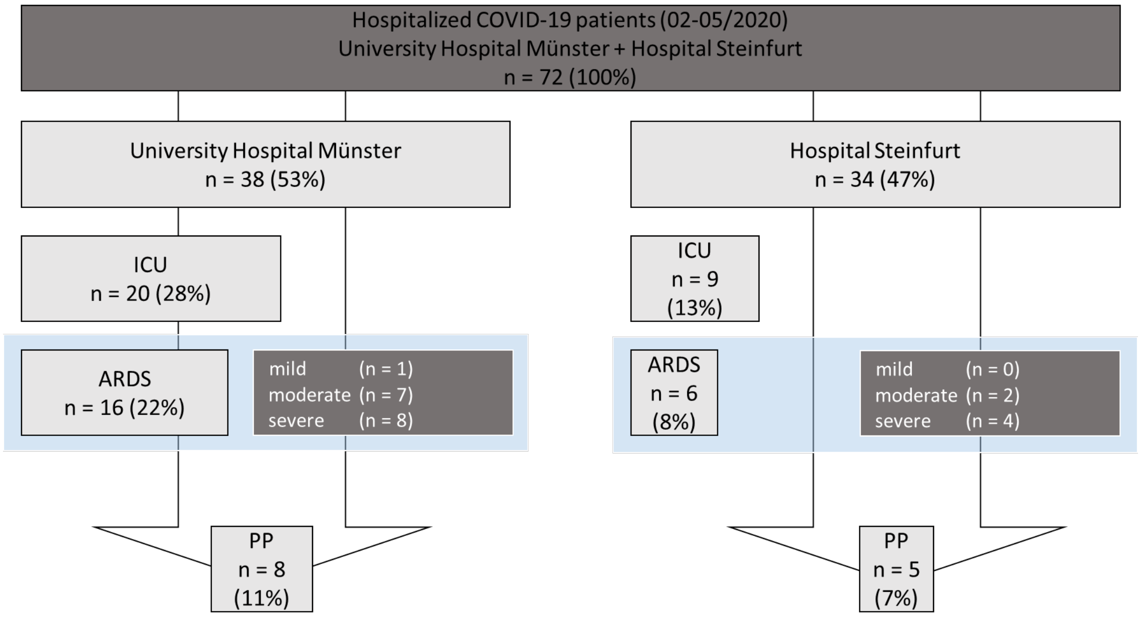

Supplement: Supplementary file 1 [file jcm-10-01046-s001.pdf]
